# State and future of radiology and nuclear medicine in Vietnam

**DOI:** 10.2349/biij.5.4.e34

**Published:** 2009-10-01

**Authors:** HD Kiet

**Affiliations:** Vietnam Radiology and Nuclear Medicine Society, Vietnam

Radiology in Vietnam was really established after Victory Dien Bien Phu in May 1954, which was the end of the war against French invasion. Along with development of health care, the system of community health care and the program of socialization of health care of Vietnam were developed, after the war against the United Statesin 1975. Radiology has grown very fast and nowadays it can meet most diagnostic imaging and nuclear medicine needs of clinical specialties.

Health care in Vietnam in general: Vietnam is an ASEAN member with an area of 331,150 km2 and a population of 85.7 million. The number of physicians is 6.25 per 10,000 people. The estimated number of hospital beds is 25/10,000 people while governmental hospital beds numbers 19/10,000 people. Medical costs for every inhabitant is VND 499,300 (USD 27) /year.

Radiology and Vietnamese Radiology and Nuclear Medicine Society in brief: after 1954, some hospitals in big cities such as Hanoi, Saigon and Hue had few X-ray diagnostic equipments for bone, respiratory, digestive system as well as paediatrics use. After the war against the United States and Liberation of Saigon in April 30, 1975, Vietnamese Radiology possessed some powered X-ray equipment ranging from 300 to 1000 mA and X-ray-TV system in hospitals, provinces and cities. In some big cities, there were angiography equipment and planar gamma camera.

**Figure 1:**
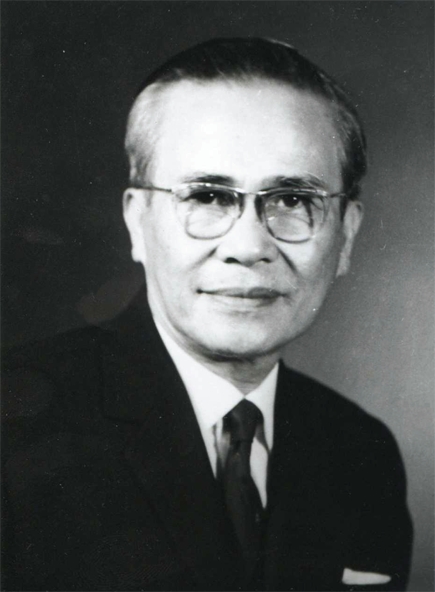
Dr. Hoang Su, the first Vietnamese radiologist

In 1983, the first ultrasonograph was done in Viet Duc Hospital, Hanoi. In 1991, the first CT scanner was installed in Viet-Xo hospital, Hanoi, where in 1997, the first superconductive MRI system was installed to serve the patients in hospitals around Hanoi. Nuclear medicine specialty was just established in 1971 in Bach Mai hospital (Hanoi) and Cho Ray hospital (Saigon).

**Figure 2:**
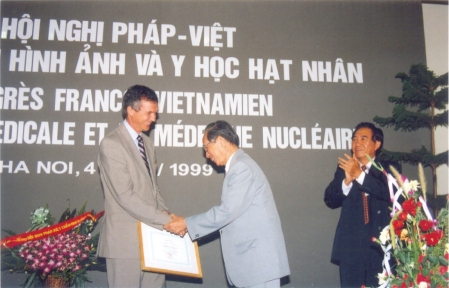
Photo from the 1st French-Vietnamese Congress on Radiology and Nuclear Medicine in 1999

With interest and investment of the Vietnam Government as well as the Ministry of Health, radiology and nuclear medicine nowadays have a network of equipment and operates from communes to districts, provincial and central level. Equipment of radiology at grassroots level includes fetal ultrasonograph, black and white ultrasonograph mode B and approximately 50% of commune infirmary have small X-ray machines. District hospitals possess X-ray and ultrasonic diagnostic equipment. About 80% of the hospitals in province, cities and all central hospitals own 174 X-ray CT scanner (including 16 to 64 MSCT and one 128 MDCT), 51 MRI systems including 16 systems 1.5 T and one machine of 3.0 T, 21 systems of angiography. Color Doppler ultrasonography is popular in provincial hospitals and some district hospitals. Some provincial and central hospitals possess angiography equipment. Regarding nuclear medicine equipment, 25 provincial hospitals and all the central hospitals have planar gamma camera and SPECT (Single photon emission computed tomography). Since 2009, there are four PET-CT and one SPECT-CT in the whole country.

**Figure 3:**
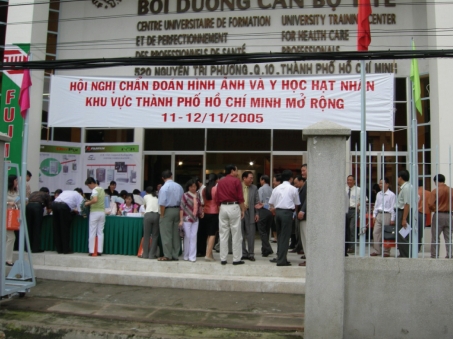
8th National Congress on Radiology and Nuclear Medicine in Ho Chi Minh City in 2005

Vietnam Radiology Society was founded in 1961 by Dr Hoang Su. He is the first specialist in radiology and was trained in France in 1940s. From 1954 to 1975, when the country was divided into two areas, activities of the Society were localized in Northern Vietnam. During 50 years of development, the Society has changed its name three times: Radiology and Physiotherapy Association (1961), Radiology-Physiotherapy- Medical Radioactivity Association (1978), and Radiology and Nuclear Medicine Society (1995). Since that time, its activities have been extended to the whole country and included three sectors: X-ray, ultrasound and nuclear medicine.

**Figure 4:**
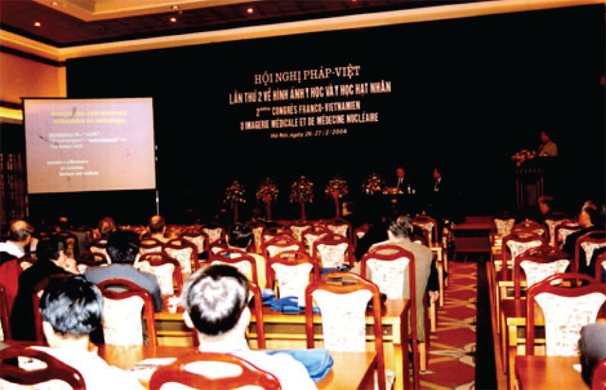
2nd French- Vietnamese Congress on Radiology and Nuclear Medicine in Hanoi

Vietnam Radiology and Nuclear Medicine Association includes more than 800 members and six-member assemblies in a number of cities and provinces, namely Ho Chi Minh, Danang, Binh Dinh, Thua Thien-Hue, Hai Phong, and Thanh Hoa.

Staff training in the field of radiology was conducted at four Universities such as: Hanoi Medical University, Ho Chi Minh City Medical University, Military Medical Academy, and Medical Faculty in Hue city. In 2008, Medical Faculty of Hai Phong City also started to train oriented X-ray physicians. Not all the radiologists are of the same level due to uneven education before. Since 2000, based on the decision of Ministry of Health to combine ultrasonography with X-ray to form Diagnostic Imaging unit in public health facilities, the department of diagnostic imaging has included ultrasound diagnosis. Nuclear specialists began to be trained from 1989 in Hanoi.

Today, diagnostic imaging physicians are trained according to three levels:

- Oriented specialists are trained within 9 months, in the field of X-ray and diagnostic ultrasonography.
- Specialists at level 1 are trained for 2 years in addition to oriented education.
- Training of specialists at level 2 (2 years) is reserved for specialist level 1 who has at least 5 years experience.
- Nuclear medicine physicians are separately trained and also follows three levels as stated above. At present, there are only two training establishments for this specialty such as Hanoi Medical University and Military Medical Academy.
- Internship physicians in diagnostic imaging began to be trained only at Hanoi Medical University from 1978. Until now, there have been 27 classes with 43 X-ray physicians graduated and assumed the key position in major hospitals and medical universities in whole country. Since 2007, Medical University in Ho Chi Minh City and Hue City have also trained resident physicians in the field of diagnostic imaging.
- Regarding diploma, oriented specialists can take an examination and enter a masters program (2 years) followed by a PhD program (3 years) after succeeding the PhD examination. To graduate, MA and PhD specialists have to complete their own thesis.
- Only three colleges in Hai Duong, Da Nang and Ho Chi Minh City train medical radiology technicians. Radiology and nuclear medicine establishments seriously lack of medical radiology technicians.

**Figure 5:**
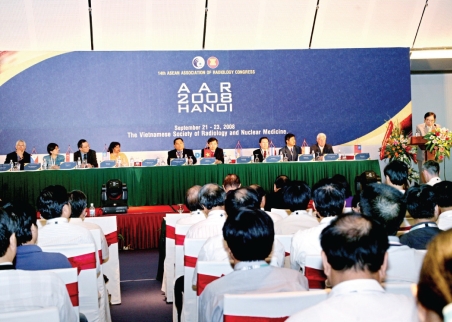
14th AAR Meeting and 10th National Congress in Hanoi in 2008

**Figure 6:**
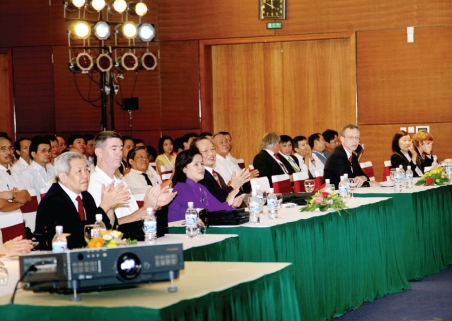
14th AAR Meeting and 10th National Congress in Hanoi

In addition to in-country training, Radiology of Vietnam has received the support of French colleagues through the cooperation between Hanoi Medical University and Vietnam Radiology Society, and the French Radiology Trainer Association. (GREF-Groupe de Radiologists Enseignements d’Expression Francaise). Every year, France sends doctors to Vietnam to give lectures and choose young radiology physicians of Vietnam to undergo a complimentary 1-year practice internship program in France. From 1994 to 2009, 114 young Vietnamese radiology physicians attended this program.

Few radiology and nuclear medicine specialists have taken a short-term course in Switzerland, the United States, South Korea, Singapore and Thailand. The purpose to develop radiology and nuclear medicine from now to 2015:

1. Organization:
  - Fully construct a national radiological center in Hanoi.
  - Fully construct a national nuclear medicine center in Hanoi.
  - In 2020, combine two centers as stated above to establish a national radiation medicine center.
2. Equipment:
  - In addition to currently available radiology equipments, it should strive to equip 80 % of provinces and cities at least one DSA machine.
  - Regarding nuclear medicine equipments, it should strive to provide one scintigraphy equipment for every 1 million people.
  - Develop the technical bimodality of PET-CT and SPECT-CT in big cities and regional provincial hospitals.
3. Training:
  - Force oriented specialists to upgrade to specialists level 1.
  - Strive to train physicians within 5 years to integrate with other countries in the region.
  - Train-the-trainer should be priority based on internship resources.
  - Continue international cooperation program in the field of radiology and nuclear medicine training to add more high-level physicians, and establish different groups of subspecialisation in radiology and nuclear medicine such as paediatrics, obstetrics, neurology, cardiovascular, digestive, respiratory and interventional radiology.
  - Enhance to train radiology and nuclear medicine technicians graduated from intermediate school and college to meet the needs of public and private health facilities.

Vietnam is a developing country, which still has lower per capita income. The development period of Vietnamese radiology was only started in 1975 . Under the open door policy of the government, like Vietnam public health, the Vietnamese radiology received several effective aids from the French Society of Radiology and colleagues from regional countries. Two French-Vietnamese Congresses on Medical Imaging and Nuclear Medicine in 1999 and 2003, and also the 14th AAR Meeting in 2008 in Hanoi were fruitful events.

To improve the national level of radiology and nuclear medicine, every effort should be concentrated on the following three main tasks:

- Expanding the investment on medical imaging and nuclear medicine equipments by all different sources.
- Carrying out the annual Continuous Medical Education Program for every radiologist of all levels.
- Realising closer cooperation with regional radiological organisations like AAR and AOCR and other developed countries to improve special knowledge and clinical experience.

